# Comparative In Vitro Evaluation of Residual Fiber Contamination from Applicators in the Adhesive Interface

**DOI:** 10.3290/j.jad.c_2687

**Published:** 2026-05-13

**Authors:** Alicia Miguel-Calvo, Santiago Berrendero, Belén Morón-Conejo, Francisco Martínez-Rus, Guillermo Pradies, Maria Paz Salido

**Affiliations:** a Alicia Miguel-Calvo Postdoctoral Assistant Professor, Universidad Complutense de Madrid, Conservative and Prosthetic Dentistry, Comunidad de Madrid, Provincia de Madrid, Madrid, Spain. Study design and concept, material preparation, and data acquisition.; b Santiago Berrendero Postdoctoral Assistan Professor, Universidad Complutense de Madrid, Conservative and Prosthetic Dentistry, Comunidad de Madrid, Provincia de Madrid, Madrid, Spain. Study design and concept, analysis, and interpretation of data.; c Belén Morón-Conejo Assistant Professor, Universidad Complutense de Madrid, Conservative and Prosthetic Dentistry, Comunidad de Madrid, Provincia de Madrid, Madrid, Spain. Study design and concept, wrote the first manuscript draft.; d Francisco Martínez-Rus Full-time Professor, Universidad Complutense de Madrid, Conservative and Prosthetic Dentistry, Comunidad de Madrid, Provincia de Madrid, Madrid, Spain. Study design and concept, material preparation, data acquisition, analysis, and interpretation of data.; e Guillermo Pradies Full-time Professor, Universidad Complutense de Madrid, Conservative and Prosthetic Dentistry, Comunidad de Madrid, Provincia de Madrid, Madrid, Spain. Study design and concept, reviewed and edited the manuscript.; f Maria Paz Salido Full-time Professor, Universidad Complutense de Madrid, Conservative and Prosthetic Dentistry, Comunidad de Madrid, Provincia de Madrid, Madrid, Spain. Study design and concept, material preparation and data acquisition, reviewed and edited the manuscript.

**Keywords:** adhesive interface, adhesive materials, application duration, contamination, dental adhesion, fibers, universal adhesives

## Abstract

**Purpose:**

To evaluate and compare the presence and distribution of residual fibers from two different brands of adhesive applicators within the adhesive interface after active application of a universal adhesive.

**Methods and Materials:**

Eighteen sound human molars were prepared with standardized Class II cavities and randomly assigned to two groups (n = 9) according to the applicator used: group P (Proclinic SAU, Spain) or group K (Kerr, USA). A one-step self-etch universal adhesive (Scotchbond Universal Plus; Solventum, USA) was actively applied following the manufacturer‘s instructions. After polymerization, specimens were examined under ultraviolet (UV) light using an optical microscope. Residual fibers were identified, quantified, and categorized according to their location (external cavity surfaces, cavosurface margins, internal line angles, axial walls, and cavity floors). Data were analyzed using the Fisher-Freeman-Halton exact test (*P* < 0.05).

**Results:**

Residual fibers were detected in all specimens, predominantly on external cavity surfaces, cavosurface margins, and axial walls. Statistically significant differences were observed between the two applicator brands (*P* < 0.05), with group P showing a higher number and greater length of fibers than group K.

**Conclusion:**

Both applicator brands released microscopic fibers that became incorporated into the adhesive interface, revealing an unrecognized source of contamination and leading to rejection of the null hypothesis. Fiber distribution was not homogeneous across cavity surfaces. The proposed methodology proved effective for detecting and localizing applicator-derived residues, highlighting an overlooked source of contamination that may influence adhesive performance and restoration durability.

Adhesion has become a fundamental principle in modern dentistry, as few clinical procedures are performed without the use of adhesive techniques. Consequently, a comprehensive analysis of the factors influencing adhesion is essential for advancing in this field. Among these factors, adhesive application techniques have been among the most extensively studied. Several studies have demonstrated that the method of adhesive application directly influences dentin bond strength,^6,9^ and its long-term stability,^7^ highlighting the importance of parameters such as application time,^9^ active application,^3,6,14,15,22,25,28^ solvent evaporation method,^5,15^ and control of adhesive layer thickness.^9,15^

Focusing on the active application of adhesives, the literature indicates that this approach enhances both bond strength and the long-term stability of the adhesive interface,^6,17,18,19,28^ as the friction generated during application increases interaction with the dental substrate and promotes deeper adhesive penetration,^2,11,17,18,24^ This effect is particularly relevant for self-etch adhesives,^20,25^ among which universal adhesives represent the most recent evolution of this bonding strategy, following the introduction of the first product of this category, Scotchbond Universal Adhesive (3M ESPE, USA), in 2012.^26^ In such systems, the transport of fresh acidic monomers to the basal portion of the smear layer can result in deeper demineralization and improved hybrid layer formation.^9,18,27^ Additionally, the motion generated during active application may facilitate solvent evaporation,^11,23^ disperse entrapped air within the adhesive, and improve the distribution of etching by-products, allowing for their more efficient removal or dispersion within the hybrid layer.^27^ Furthermore, several studies have suggested that active application may even improve dentin bond strength in cases where the adhesive interface becomes contaminated with saliva.^3^

Active application requires the use of an appropriate applicator capable of exerting effective rubbing on the tooth surface, and microbrushes have been shown to be the most suitable for this purpose.^13,23^ These devices consist of a plastic handle with an articulating joint that facilitates access to the desired area of the tooth,^12^ and an active tip composed of non-absorbent nylon microfibers attached to the end of the handle. During use, fibers or bristles from this flocked portion may detach and become incorporated into the adhesive interface, potentially acting as contaminants. In fact, in an observational study, Berton et al^4^ detected the presence of fiber remnants in 100% of the cavities examined using scanning electron microscopy (SEM), suggesting a possible interference with adhesion and restoration durability. Similar findings were recently reported by Padwal et al,^21^ who also used SEM to evaluate dental surfaces on which two different adhesives (one etch-and-rinse and one self-etch) had been applied with the same type of applicator. Although numerous studies have investigated contamination of the adhesive interface by various agents,^7^ aside from these two reports, there is surprisingly no other evidence in the literature addressing contamination caused by fibers detached from adhesive applicators.

Therefore, the present study aimed to develop a method to detect and localize fiber remnants from adhesive applicators within the adhesive interface. The null hypothesis (H0) stated that no differences would be observed in fiber detachment between two different applicator brands following the active application of a universal adhesive in standardized dental cavities. Accordingly, the objective was to evaluate and compare the presence and distribution of fiber remnants resulting from the use of two commercially available applicators during an adhesive procedure.

## MATERIALS AND METHODS

### Study Design

**Fig 1a and b:**
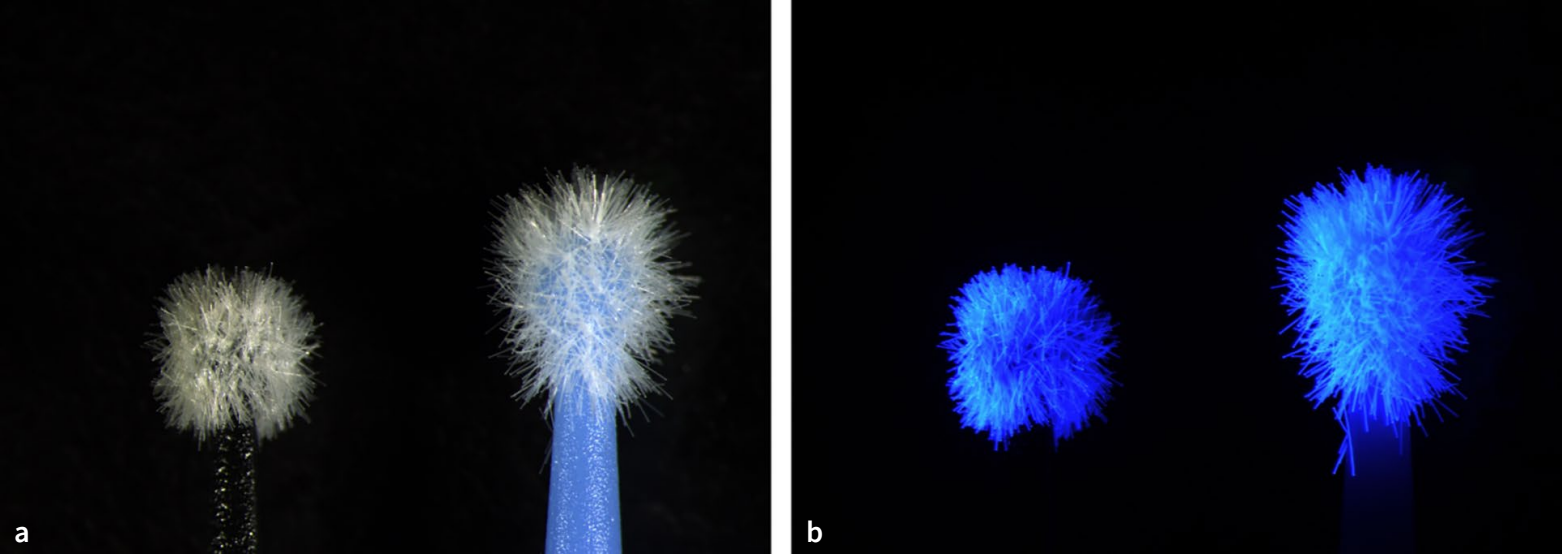
(a) Applicators evaluated under normal lighting conditions: on the left, the Kerr applicator (Kerr, USA); on the right, the Proclinic applicator (Proclinic SAU, Spain). (b) Applicators evaluated under ultraviolet (UV) lighting conditions: on the left, the Kerr applicator (Kerr, USA); on the right, the Proclinic applicator (Proclinic SAU, Spain).

This was an *in vitro*, observational, and comparative study designed to evaluate the presence of fiber remnants within the adhesive interface after the use of an adhesive system applied with applicators from two different commercial brands: Proclinic (Proclinic Micro Applicator, Proclinic SAU, Spain) and Kerr (Kerr Applicators, Kerr, USA). These applicators were selected because their bristles exhibit fluorescence under ultraviolet (UV) light, facilitating their identification when examined under this type of illumination (Fig 1). It is noteworthy that the Kerr group represents a well-established brand with extensive experience in adhesive dentistry, whereas Proclinic belongs to a product line from a major distributor, offering a more “private-label” applicator system.

### Sample Collection: Natural Teeth

A total of eighteen sound human molars, free of caries and restorations, were collected. The teeth were extracted for periodontal or orthodontic reasons from patients over 18 years of age who attended the General and Postgraduate Clinics of the Faculty of Dentistry at the Complutense University of Madrid (UCM). All patients provided written informed consent prior to tooth donation.

To ensure sample standardization, teeth that were restored, exhibited visible fractures or cracks, or presented damaged crowns or carious lesions were excluded from the study. Teeth from patients who declined to participate were also excluded.

The selected teeth were manually cleaned using ultrasonic instruments (Acteon Newtron P5 XS B.LED, Acteon, France), disinfected, and stored at 4ºC in Ringer’s solution prepared by the Research Laboratory of the Faculty of Dentistry, UCM, following the formulation described by Heck et al.^16^

### Tooth Positioning and Experimental Procedure

Each tooth was fixed by its root into a resin block (EU High Impact Denture Base resin, Sprintray, USA) embedded within a 20 × 20 × 20 mm methacrylate cube, exposing only the coronal portion. The specimens were subsequently stored under the same conditions described above.

In each molar, a Class II cavity with divergent walls was prepared. The occlusal box measured approximately 2 mm in depth and 2 mm in width, while the proximal box measured 4 mm in depth with a cavity floor of 3 × 3 mm. Cavity preparations were performed using a flat-end tapered diamond bur with coarse grit (ISO 806 314 546547 018, Komet Dental, Germany) at high speed (500,000 rpm) under copious water irrigation. Cavity dimensions were verified using a graduated periodontal probe (model PCP UNC156, Hu-Friedy, USA). All cavity preparations were performed by a single experienced operator under 4× magnification (Ergo 4× Vittrea, IPG Dental Group, Spain).

### Adhesive Application Procedure and Group Allocation

All teeth and prepared cavities were initially examined under an optical microscope (M-125, Leica, Germany) at 7.5× magnification, first under white light (CLS 100 LED, Leica Microsystems, Germany) and subsequently under ultraviolet (UV) illumination using two 365 nm, 10 W light sources (Alonefire UV, Shenzhen Shiwang Lighting, China). This preliminary inspection ensured the absence of fluorescent residues that could be mistaken for applicator remnants.

The eighteen specimens were randomly divided into two experimental groups:

Group P (n = 9): Adhesive procedures were performed using Proclinic applicators (Proclinic SAU, Spain) obtained from three different manufacturing batches. Three applicators from each batch were used on three randomly selected samples, ensuring equal distribution of the batches among the nine specimens.
Group K (n = 9): Adhesive procedures were performed using Kerr applicators (Kerr, USA), also obtained from three distinct batches and distributed in the same manner as in Group P.

To simulate clinical conditions, each cavity was encircled with a circular matrix band (Automatrix, Dentsply Sirona, USA). After rinsing and drying, a one-step self-etch universal adhesive (Scotchbond Universal Plus, Solventum, USA) was applied strictly according to the manufacturer’s instructions. The adhesive was actively rubbed onto the cavity surface with the applicator for 20 s, followed by a gentle stream of air for at least 5 s until a uniform, glossy surface was obtained. Polymerization was performed with a Valo Cordless curing light (Ultradent Products, USA) for 20 s in the occlusal cavity and an additional 20 s in the proximal cavity. All adhesive procedures were carried out by a single trained operator to ensure procedural consistency.

### Microscopic Analysis

**Fig 2:**
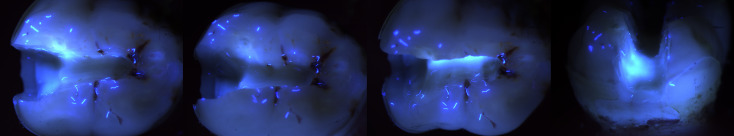
Photographic sequence under ultraviolet (UV) light showing the four examined perspectives of a sample from the K group (Kerr, USA): occlusal, 45° inclined, and proximal views, with fluorescent residual fibers visible across different areas of the cavity.

After completion of the adhesive procedure, the matrix was removed, and the teeth were re-examined using the same microscope employed for the preliminary inspection, this time under UV illumination only. Photographs were obtained, and each specimen was examined from four perspectives: occlusal view, at 45º inclinations toward the buccal and lingual aspects, and proximal view (Fig 2). When necessary, multiple images at different focal depths were captured from the same perspective, as well as from additional angles when required.

**Fig 3:**
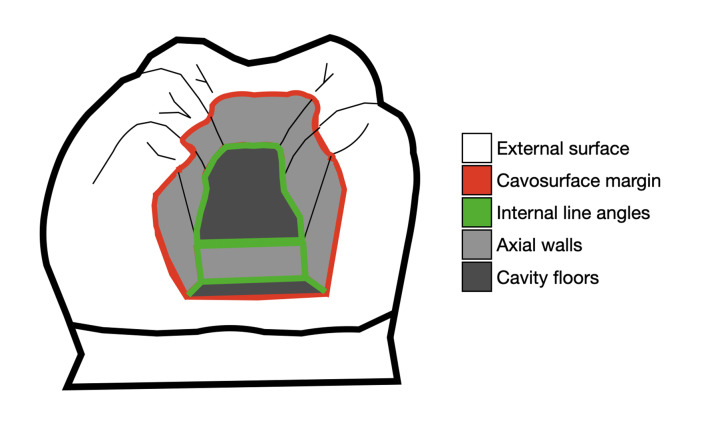
Potential locations of applicator fiber remnants within the tooth: external surfaces, at the cavity margins, on the axial walls, on the cavity floor, and in the internal line angles.

Images were captured using a camera coupled to the microscope (DFC450, Leica, Germany) to assess the presence or absence of fiber remnants from the applicators and to determine their spatial distribution. Five positions were distinguished: external cavity surface, cavosurface margins, internal line angles, axial walls, and cavity floors (Fig 3).

Fiber counting was independently performed by two blinded and previously calibrated operators (Cohen’s kappa > 0.80). To quantify the results, five ordinal categories were established: 0, no fibers observed; A, 1–3 fibers; B, 4–6 fibers; C, 7–9 fibers; and D, ≥ 10 fibers.

### Statistical Analysis

Data analysis was performed using IBM SPSS Statistics software (version 29.0.2.0; IBM, USA). Descriptive statistics were calculated to summarize the distribution of fiber remnants across the different cavity locations and applicator types. For inferential analysis, the Fisher-Freeman-Halton exact test was applied to assess the presence of statistically significant differences in the frequency distribution of fiber categories between the two applicator brands and among cavity surfaces. The level of statistical significance was set at *P* < 0.05.

## RESULTS

**Fig 4:**
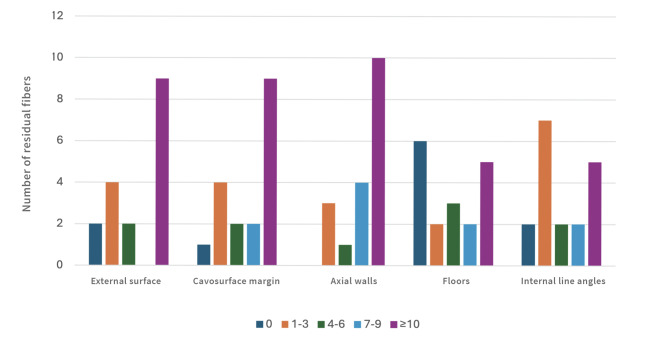
Distribution of samples according to the range of residual fibers detected on the different cavity surfaces.

Descriptive analysis revealed the presence of residual fibers on all examined cavity surfaces. The highest number of fibers was observed on external cavity surfaces, at the cavosurface margins, and along the axial walls, whereas the cavity floor and internal line angles exhibited the lowest counts (Fig 4).

The Fisher-Freeman-Halton exact test indicated statistically significant differences in the distribution of residual fibers among the different cavity surfaces (*P* < 0.001).

**Fig 5:**
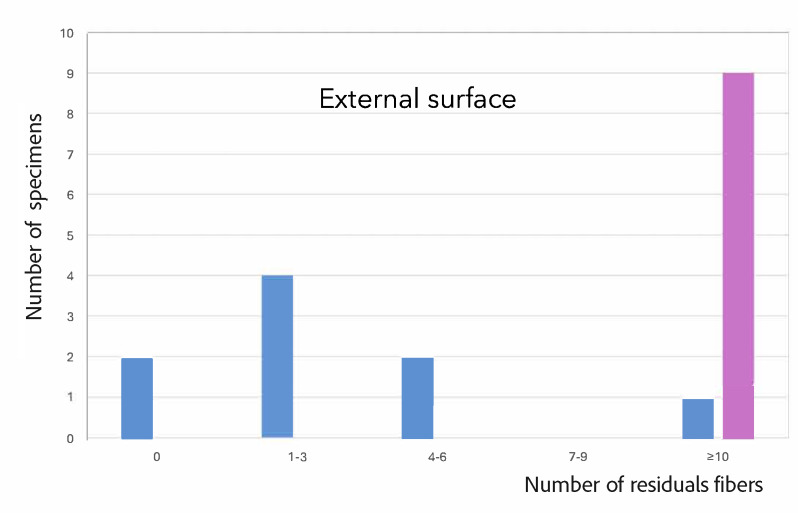

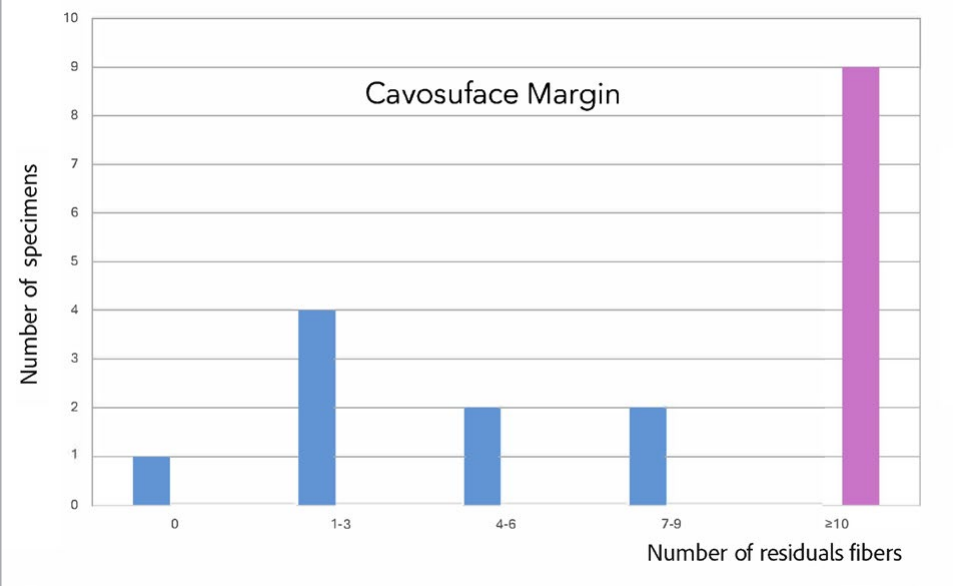

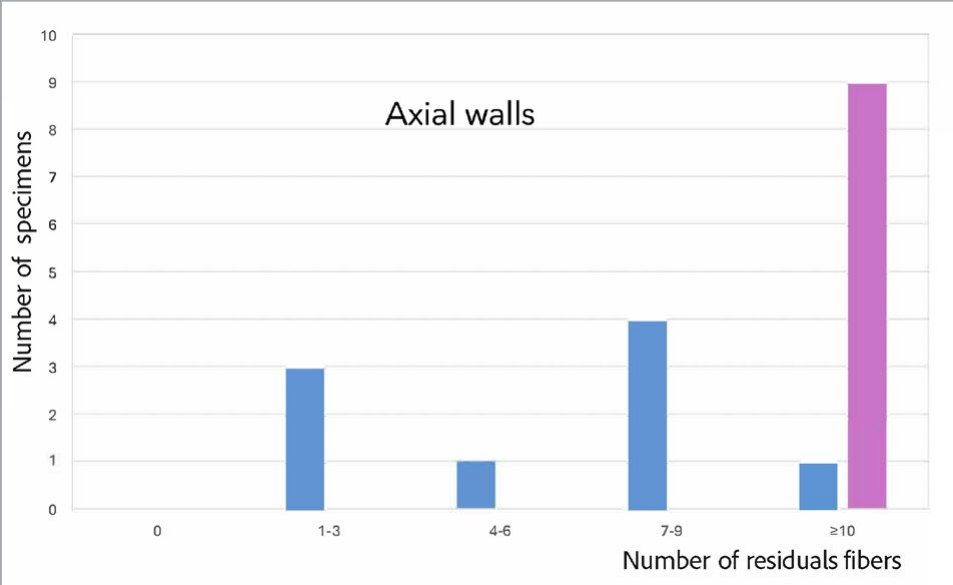

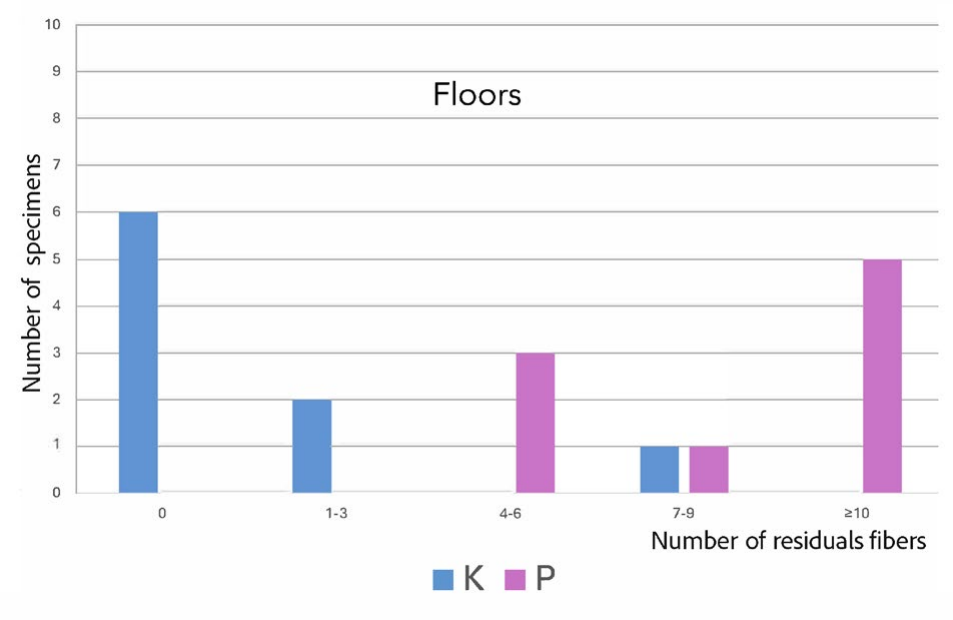

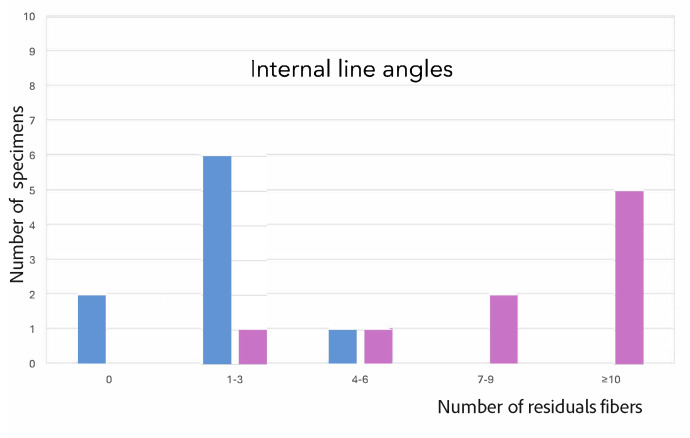
Distribution of samples according to the number of residual fibers detected on each cavity surface (external surface, cavosurface margin, axial walls, cavity floors, and internal line angles), differentiated by applicator type (Kerr – K; Proclinic – P). The vertical axis represents the number of specimens, and the horizontal axis indicates the range of residual fibers detected.

When analyzed by applicator brand, group P exhibited a greater number of residual fibers than group K across all cavity surfaces (Fig 5). On the external surface, cavosurface margin, and axial wall, all P samples presented ≥ 10 fibers (category D), with no samples showing lower counts. Conversely, group K applicators left fewer residues overall, particularly at the internal line angles, where two samples presented no fibers (category 0), six showed 1–3 fibers (category A), and one showed 4–6 fibers (category B).

Application of the Fisher-Freeman-Halton exact test confirmed statistically significant differences (*P* < 0.05) between the two applicator brands for all cavity surfaces (Table 1).

**Table 1:** Results of the Fisher-Freeman-Halton exact test by brand and surface area

Cavity surface	Fisher–Freeman–Halton test value	Exact sig. (2-sided)
External surface	13,439	< 0.001
Cavosurface margin	17,698	< 0.001
Axial walls	13,727	< 0.001
Floors	15,395	< 0.001
Internal line angle	11,755	0.006


## DISCUSSION

The results of the present study demonstrate that both applicator brands left fiber remnants within the adhesive interface, thereby rejecting the null hypothesis, as statistically significant differences were observed in the number of detached fibers between the two brands.

Numerous studies have investigated contamination of the adhesive interface by saliva and blood,^27^ and even the effects of other contaminants such as water, residual hemostatic or topical anesthetic agents, oil from rotary instruments or air compressors, glove powder, and cleaning agents from dental unit waterlines.^7^ However, interestingly, apart from the studies by Berton et al^4^ and Padwal et al,^21^ which reported the presence of fiber remnants originating from adhesive applicators, no other investigations have specifically addressed this form of contamination within the adhesive interface.

In the aforementioned studies, SEM analyses performed after completion of the adhesive procedure revealed the presence of fibers originating from the applicators in all examined specimens. However, to date, the potential impact of these remnants on the quality and durability of the adhesive interface has not been specifically investigated. Nevertheless, Alshawi et al (2024) suggested that the type of adhesive applicator may influence bonding performance. In their study, a higher percentage of adhesive failures and lower stress at failure were observed in samples where the adhesive was applied using microbrush applicators compared with those in which a bristle brush was used.^1^ However, the authors did not verify the presence or absence of applicator-derived fibers at the adhesive interface, which could potentially explain the different bonding behavior observed between the applicator types.

In the absence of studies reporting the consequences of the presence of applicator residues on the adhesive interface, Berton et al^4^ postulated that residual fibers could interfere with bond strength, marginal sealing, and the polymerization behavior, thereby favoring the formation of microgaps within the adhesive interface. This assumption appears plausible, as the interposition of fibers may impede intimate contact between the adhesive and the dental substrate, resulting in the formation of localized areas of incomplete interaction (“shadow zones”) at the interface. In etch-and-rinse adhesive systems, such zones may correspond to demineralized dental tissue that remains uninfiltrated by the adhesive, whereas in self-etch systems, they may leave the smear layer unmodified and not adequately penetrated. The presence of these adhesive “shadow zones” would effectively reduce the effective bonded surface area and introduce microstructural defects within the adhesive layer, which could adversely affect the resulting bond strength.

It is further conceivable that the clinical relevance of such residues depends on their amount, size, and spatial distribution within the adhesive interface. However, the experimental approaches adopted in the available studies limit the assessment of these parameters: SEM analyses, used by Berton et al,^4^ do not allow continuous evaluation of the entire bonded surface, while the study of Padwal et al^21^ was conducted on flat dental substrates rather than within prepared cavities. In contrast, the methodology employed in the present investigation enabled a comprehensive examination of the tooth and the cavity surfaces following adhesive application, allowing both the quantification of detached applicator fibers and the precise determination of their localization within the cavity.

A descriptive analysis of the observations from the present study revealed a higher prevalence of residual applicator fibers on the external surface of the cavity, at the cavosurface margin, and along the axial walls, compared with the cavity floor and internal line angles (Fig 4). This distribution pattern may be attributed to the combined effects of the applicator design and procedural dynamics. During active adhesive application, friction is likely to promote more frequent fiber contact with the cavity walls than with the floor. In addition, the air stream applied for solvent evaporation may displace detached fibers outward from the inner cavity, favoring their accumulation along the walls, cavosurfaces margins, and external surfaces. If, as suggested by Berton et al,^4^ residual fibers are capable of promoting the formation of microgaps within the adhesive interface, and considering that the presence of microgaps is particularly detrimental when located at the cavosurface margin, the finding of the present study raises concerns regarding the potential long-term implications of applicator-derived fiber contamination. Specifically, the preferential localization of residual fibers at the cavosurface margin suggests that this form of contamination may compromise marginal integrity over time, an area known to be critical for the clinical longevity of adhesive restorations.

**Fig 6a and b:**
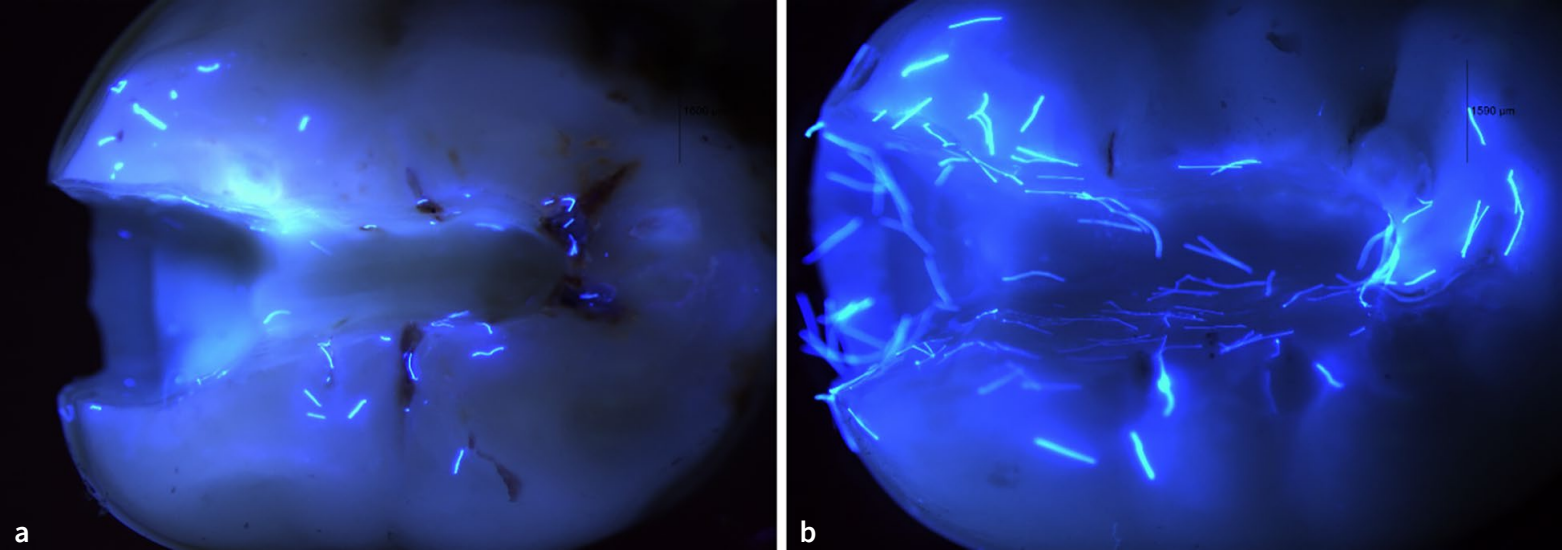
Comparison of residual fiber counts between study groups: (a) Kerr (K) applicators showed fewer remnants, particularly at the internal line angles. (b) Proclinic (P) applicators consistently left a greater number of fibers across all cavity surfaces.

When comparing the two applicator brands tested, statistically significant differences (*P* < 0.05) were observed across all examined cavity surfaces (Table 1). Brand P exhibited a consistently higher overall number of residual fibers (Fig 5). On the external surface, cavosurface margin, and axial walls, all specimens in this group presented ≥ 10 fibers, with no samples showing lower counts. In contrast, brand K left fewer fiber remnants, particularly in the internal line angles, where no specimen exhibited more than 4–6 fibers. Furthermore, macroscopic examination revealed differences in fiber morphology, with fibers detached from the P-brand applicators appearing longer than those from the K-brand (Fig 6). In light of the previously discussed hypothesis, it is reasonable to assume that any potential adverse effects associated with an applicator-derived fiber contamination would be exacerbated when a greater number of fibers and/or larger fiber dimensions are present within the adhesive interface.

Beyond their potential influence on adhesive performance, the presence of detached applicator fibers may also be considered from the perspective of microplastic exposure.^9,12^ In this context, the findings of the present study suggest that adhesive applicators may represent an additional, previously unrecognized source of localized microplastic contamination within the oral environment. Although the present investigation did not assess biological interactions or systemic exposure, the repeated clinical use of fiber-based applicators raises questions regarding cumulative exposure over time. These considerations further emphasize the relevance of minimizing unnecessary contamination of the adhesive interface and highlight the need for future studies to explore the biological behavior and potential clinical implications of applicator-derived microfibers.^12^

The described method proved effective for detecting fibers detached from adhesive applicators and for determining their number, size, and location on the tooth surface. Nevertheless, several limitations should be acknowledged. First, as the method relies on optical microscopy, fiber remnants smaller than the resolution limit of the system may have gone undetected. Although both applicators evaluated in the present study displayed this behavior, manufacturer-provided information regarding fiber composition was limited. Specifically, the manufacturer of brand K does not disclose the synthetic polymer used for fiber fabrication, whereas the manufacturer of brand P only reports that the fibers are composed of polyamide. Considering that synthetic polymer fibers are not inherently fluorescent under UV illumination, and that fluorescence is typically associated with polymer impurities and/or added dyes, as demonstrated in recent spectroscopic studies (Froelich et al., 2024), this uncommon optical behavior further highlights the paucity of compositional information provided by manufacturers.^13^ Differences in fluorescence and optical response among applicators likely reflect underlying variations in material composition, which remain largely undisclosed.

Another limitation arises when samples contain a high number of residual fibers, as observed particularly with the P-brand applicators, which render accurate counting extremely challenging. For this reason, the ≥ 10 category was introduced; however, this approach inevitably complicates the statistical interpretation of the results. An additional methodological constraint concerns the lack of measurement and control of the pressure applied during active adhesive rubbing. As reported by Reis et al,^23^ the vigor of adhesive application depends largely on the operator’s subjective perception and is inherently difficult to standardize. In the present study, all adhesive procedures were performed by a single operator under simulated clinical conditions, maintaining a constant application time of 20 s but without quantifying the applied pressure. Although this may be considered a methodological limitation, it also reflects routine clinical practice, where such control is not routinely feasible.

Furthermore, it should be noted that this study was conducted under *in vitro* conditions. Therefore, potential differences in the behavior of both the adhesive system and the applicators under intraoral conditions cannot be excluded, which limits direct clinical extrapolation of the findings. In addition, the limited amount of previous research specifically addressing applicator-derived fiber contamination restricts direct comparison with other studies and the broader contextualization of the present results.

Finally, an important limitation of this study is that the results only demonstrate the presence of applicator-derived residues at the interface, with varying amounts depending on the applicator used and with non-homogeneous distribution throughout the cavity. However, the influence that these residues may have on both immediate and long-term adhesive performance remains unknown. Therefore, further studies are required to clarify the potential clinical implications of these findings.

## CONCLUSIONS

Within the limitations of this *in vitro *study, both adhesive applicator brands evaluated released fibers that became incorporated into the adhesive interface. Significant differences were observed between the two brands, with group P applicators producing a higher number and greater length of residual fibers compared with group K applicators. These results demonstrate that detachment and retention of applicator-derived fibers during active adhesive applications is a consistent and previously underrecognized phenomenon. Further studies are warranted to investigate the clinical relevance of these findings and to determine the potential impact of such residues on adhesive interfaces under functional conditions.

### Acknowledgments

#### Clinical relevance

Microscopic fiber residues originating from applicators were detected within the adhesive interface, revealing an unrecognized source of contamination. These findings underscore the importance of applicator quality and the use of controlled adhesive application to achieve reliable bonding.

#### Conflict of interest

The authors report no conflicts of interest related to this study and it was self-funded.
